# Tracking a Killer: In Vivo Microscopy Reveals Details on the Life Cycle of Malarial Parasites

**DOI:** 10.1371/journal.pbio.0030215

**Published:** 2005-05-24

In a perfect world, anyone frequenting the habitat of malaria-carrying mosquitoes would sleep beneath a mosquito net, take prophylactic drugs, and maybe even quaff quinine-laced tonic water. But life is rarely perfect and even though governments and the World Health Organization have combined such prevention measures with widespread efforts to eradicate malaria-carrying mosquitoes, the disease kills more than a million people each year.

The microbes that cause the disease, tiny parasites of the genus *Plasmodium*, have a complex life cycle that involves several distinct phases and habitats. When a person is bitten by an infected mosquito, highly mobile *Plasmodium* cells (called sporozoites) migrate from the skin into the bloodstream, which carries them to the liver, where they set up shop in liver cells (called hepatocytes) and multiply asexually as merozoites. Eventually the parasites leave the liver and reenter the bloodstream, where they invade red blood cells, multiply again, and differentiate into new merozoites. Ultimately, a red blood cell will become so chock-full of merozoites it bursts, releasing more merozoites to infect other red blood cells. Some of these merozoites will differentiate into male and female sex cells (called gametocytes) that hitchhike along with red blood cells when a new mosquito takes a blood meal from an infected person. The gametocytes then breed within the mosquito and produce sporozoites, which reside in the insect's gut—and then the cycle begins all over again.

Although much is already known about the *Plasmodium* life cycle, many details—including the discrete steps that facilitate sporozoites' invasion of the liver—have escaped direct observation until now. In a new study, Ute Frevert et al. literally take a closer look at this process with intravital microscopy—which allows direct observation of cell movement in a living animal—to see how the parasites gain entry into the liver.[Fig pbio-0030215-g001]

**Figure pbio-0030215-g001:**
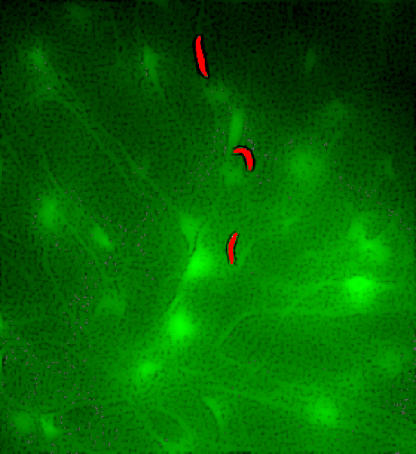
Tagged with red fluorescent protein, the malarial parasites in the sporozoite stage can be seen migrating along the sinusoids in a mouse liver

To visualize this process, the authors used genetically engineered *Plasmodium* parasites to express fluorescent tags. They introduced these fluorescent parasites to mice and rats the natural way—through mosquito bites—and then watched for the arrival of the parasites in the livers of the test animals. They observed that parasites were carried into the liver by the bloodstream, and then attached to the sinusoidal walls (sinusoids take the place of capillaries in the liver). They watched as the parasites crawled along the interior of the sinusoids—sometimes against the direction of blood flow—until they reached a specialized cell called a Kupffer cell. These star-shaped cells line the liver sinusoids and clean the blood of particulate debris and dead blood cells. But they also, apparently, serve as the parasites' portal of entry into the liver.

Earlier work had suggested that sporozoites might use Kupffer cells to access the liver, but Frevert et al. watched sporozoites traverse Kupffer cells to reach the liver interior. They observed some interesting details in this process: as sporozoites enter a Kupffer cell, they first pause, then undergo a slow constriction as they insinuate their way through the cell (rather like a napkin being drawn through a napkin ring). Sporozoites traverse the Kupffer cell at a speed much slower than they could crawl, indicating that this traversal involves more than mere parasite locomotion. Upon exiting the Kupffer cell on the other side, the sporozoites wreaked havoc in the liver, leaving a path of destruction and dead cells behind them as they moved through several consecutive hepatocytes before finally settling down in one to begin reproducing.

Frevert et al. had to make multiple attempts and track several different sporozoites at each stage in order to gain a comprehensive picture of this part of the parasite's life cycle. But thanks to the visualization advantages provided by using the fluorescent parasites and intravital microscopy, the authors show that it is now possible to directly observe events in the *Plasmodium* life cycle that had only been inferred before.

